# Supplementary material for: Prevalence of Worldwide Neonatal Calf Diarrhoea Caused by Bovine Rotavirus in Combination with Bovine Coronavirus, Escherichia coli K99 and Cryptosporidium spp.: A Meta-Analysis
Source: Animals (Basel). 2021 Apr 3;11(4):1014. doi: 10.3390/ani11041014 (PMC8066230; doi:10.3390/ani11041014)
Supplement: Supplementary file 1 [file animals-11-01014-s001.pdf]

Supplementary Material

Prevalence of Worldwide Neonatal Calf Diarrhoea Caused by Bovine Rotavirus in Combination with Bovine Coronavirus, *Escherichia Coli* K99 and *Cryptosporidium spp.*: A Meta-Analysis

Michael Brunauer [a], Franz-Ferdinand Roch [a], Beate Conrady [a,b,c*]

[a] *Institute of Food Safety, Food Technology and Veterinary Public Health, University of Veterinary Medicine, 1210 Vienna, Austria*

[b] *Department of Veterinary and Animal Sciences, Faculty of Health and Medical Sciences, University of Copenhagen, 1870 Frederiksberg C, Denmark*

[c] *Complexity Science Hub Vienna, Josefstädter Straße 39, A-1080, Austria*

* Corresponding author.
E-mail address: bcon@sund.ku.dk (B. Conrady)

Supplementary Material I

Detail description of the methodology

The mean prevalences were weighted (w_i) based on the inverse of within-study variance (v_i) and the variability across the studies(τ^2) [1], according to the Paule and Mandel method based on the formula [2,3]:

$$w_i=1/(v_i+\tau^2) \quad (1)$$

For variance-stabilization of the prevalence data distribution, Freeman-Tukey double arcsine transformation (P_i^{FT}) [4] was used, while e_i is the number of events in each $study_i$ and n_i shows the number of observations:

$$P_i^{FT} = 0.5\ (arsin\sqrt{\frac{e_i}{n_i+1}} + arsin\sqrt{\frac{e_i+1}{n_i+1}}) \quad (2)$$

The corresponding back-transformation was conducted according to the approach by Miller [5,6]:

$$P_i^{FT} = 0.5\ (1 - sin(cos(0_i^{FT}))\sqrt{1 - (sin(20_i^{FT}) + [sin(20_i^{FT}) - 1/sin(20_i^{FT})]/\tilde{n})^2} \quad (3)$$

hereby $\tilde{n}$ represented the harmonic mean of the sample size of studies I and its defined as follows:

$$\tilde{n} = I \Big/ \sum_{i=1}^{I} \frac{1}{n_i} \quad (4)$$

The Loess algorithm was applied with the locally weighted regression smoothing function and robust weightings implemented in R with the function geom_smooth and the corresponding method “loess”[7].

Reference of the Supplementary Material

(1) Scharnböck, B.; Roch, F.-F.; Richter, V.; Funke, C.; Firth, C. L.; Obritzhauser, W.; Baumgartner, W.; Käsbohrer, A.; Pinior, B. A meta-analysis of bovine viral diarrhoea virus (BVDV) prevalences in the global cattle population. *Sci. Rep.* **2018**, *8*, 1–15. https://doi.org/10.1038/s41598-018-32831-2.

(2) Paule, R. C.; Mandel, J. Consensus values, regressions, and weighting factors. *J. Res. Natl. Inst. Stand. Technol.* **1989**, *94*, 197–203. https://doi.org/10.6028/jres.094.020.

(3) Veroniki, A. A.; Jackson, D.; Viechtbauer, W.; Bender, R.; Bowden, J.; Knapp, G.; Kuss, O.; Higgins, J. P. T.; Langan, D.; Salanti, G. Methods to estimate the between-study variance and its uncertainty in meta-analysis. *Res. Synth. Methods* **2016**, *7*, 55–79. https://doi.org/10.1002/jrsm.1164.

(4) Freeman, M. F.; Tukey, J. W. Transformations related to the angular and the square root. *Ann. Math. Stat.* **1950**, 607–611.

(5) Miller, J. J. The inverse of the Freeman – Tukey double arcsine transformation. *Am. Stat.* **2012**, *32*, 3–4. https://doi.org/10.1080/00031305.1978.10479283.

(6) Schwarzer, G.; Chemaitelly, H.; Abu-Raddad, L. J.; Rücker, G. Seriously misleading results using inverse of Freeman-Tukey double arcsine transformation in meta-analysis of single proportions. *Res. Synth. Methods* **2019**, *10*, 476–483. https://doi.org/10.1002/jrsm.1348.

(7) Cleveland, W. S. Robust locally weighted regression and smoothing scatterplots. *J. Am. Stat. Assoc.* **1979**, *74*, 829–836. https://doi.org/10.1080/01621459.1979.10481038.

Supplementary data

Fig. S1: Funnel plot and influential case diagnostic for studies covering a) BRV-BCoV mixed infections, b) BRV-ETEC mixed infections and c) BRV-Crypto mixed infections. N.B. no outliers were identified for all mixed infections.

Fig. S2: Forest plot of studies with BRV-BCoV prevalences ordered by health status of the calves and publication year. N.B. The full references are provided at the end of the Supplementary Material.

Fig. S3: Forest plot of studies with BRV-ETEC prevalences ordered by health status of the calves and publication year. N.B. The full references are provided at the end of the Supplementary Material.

Fig. S4: Forest plot of studies with BRV-Crypto prevalences ordered by health status of the calves and publication year. N.B. The full references are provided at the end of the Supplementary Material.

Fig. S1: Funnel plot (left side) and influential case diagnostic (right side) for studies covering a) BRV-BCoV mixed infections, b) BRV-ETEC mixed infections and c) BRV-Crypto mixed infections. N.B. no outliers were identified for all mixed infections.

a)

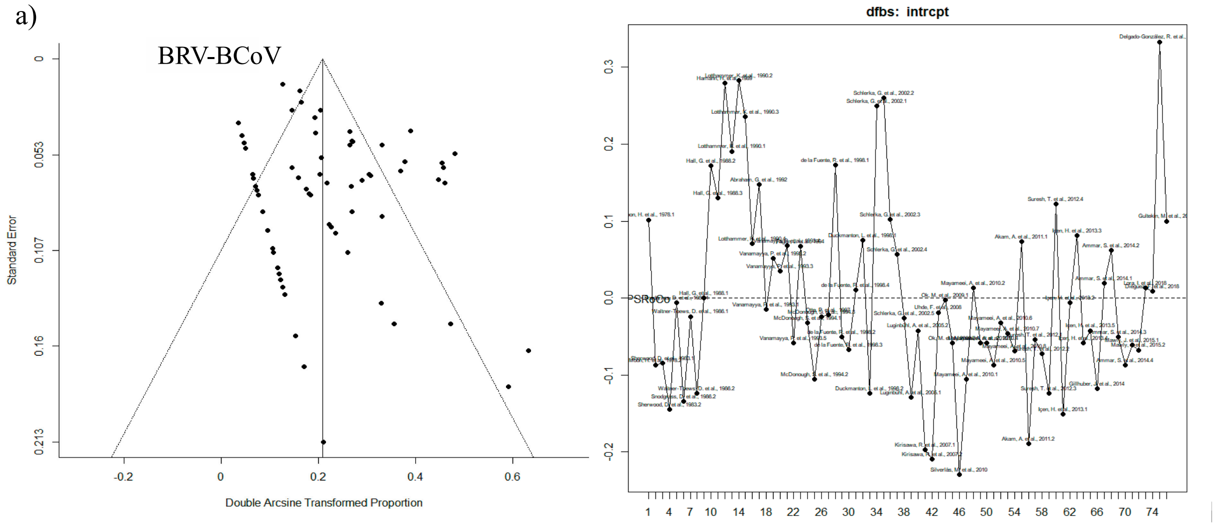

b)

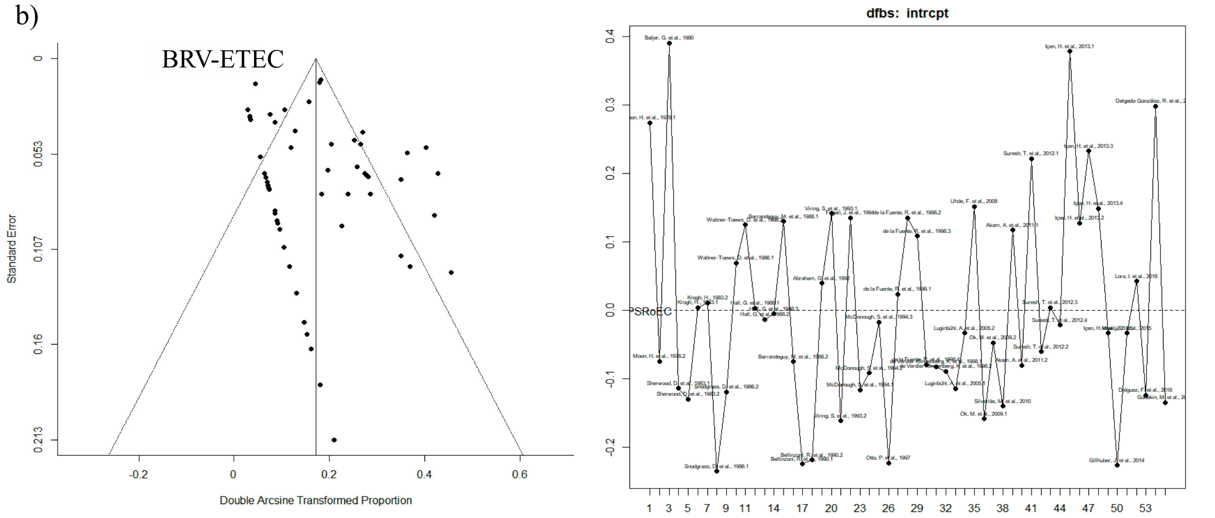

c)

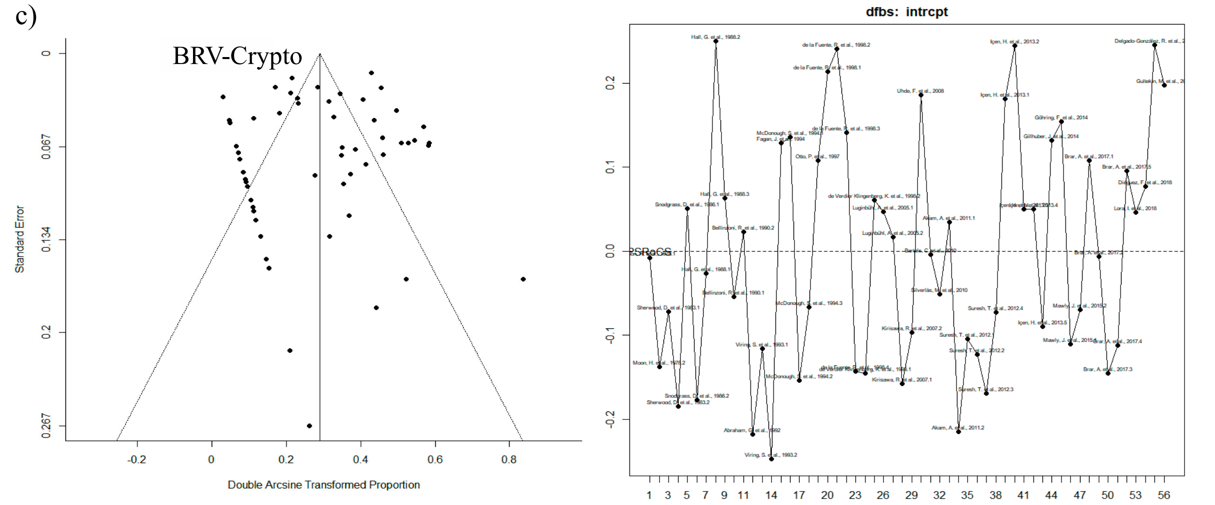

Fig. S2. Forest plot of studies with BRV-BCoV prevalences ordered by health status of the calves and publication year. N.B. The full references are provided at the end of the

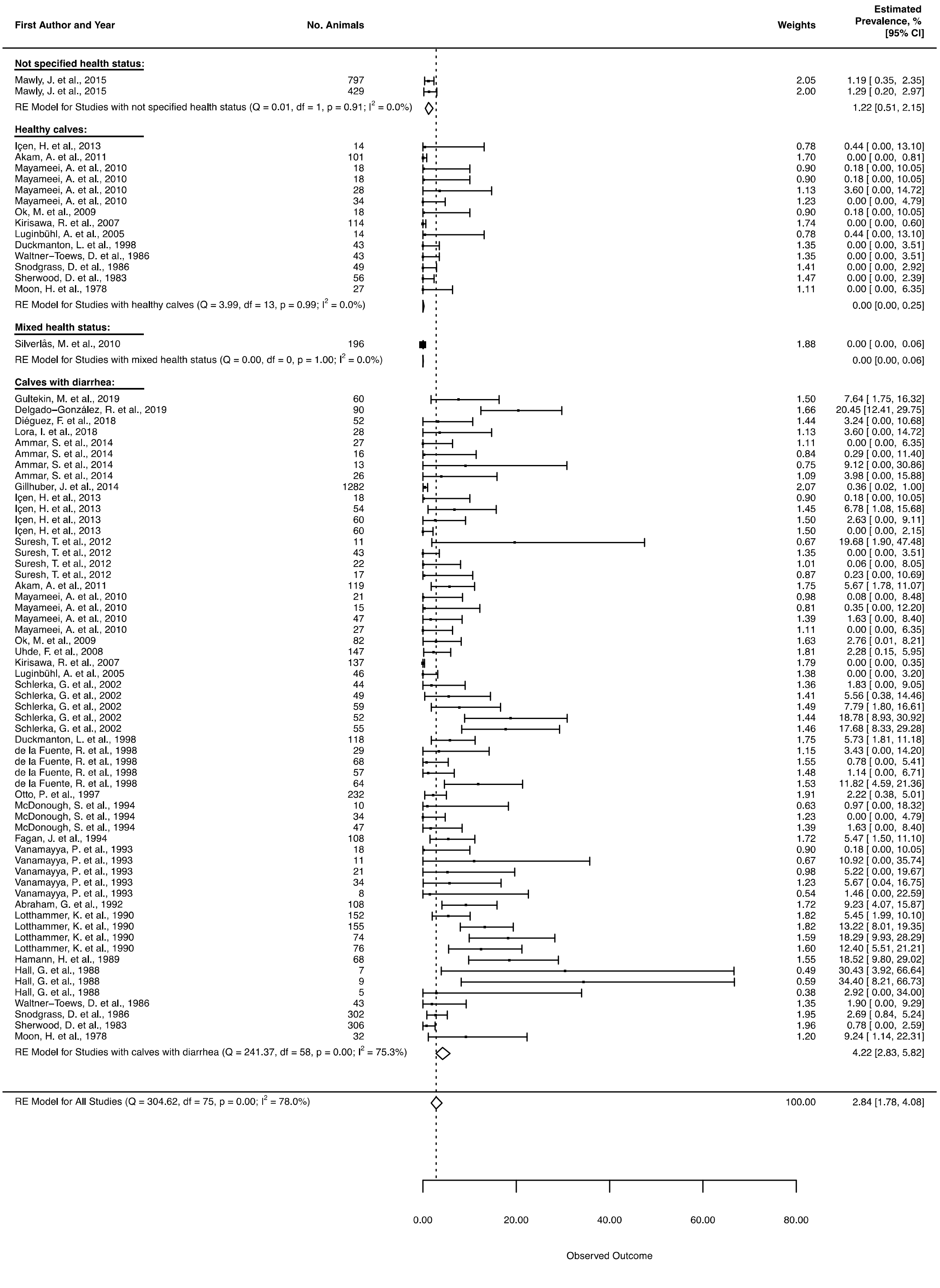

Fig. S3. Forest plot of studies with BRV-ETEC prevalences ordered by health status of the calves and publication year. N.B. The full references are provided at the end of the

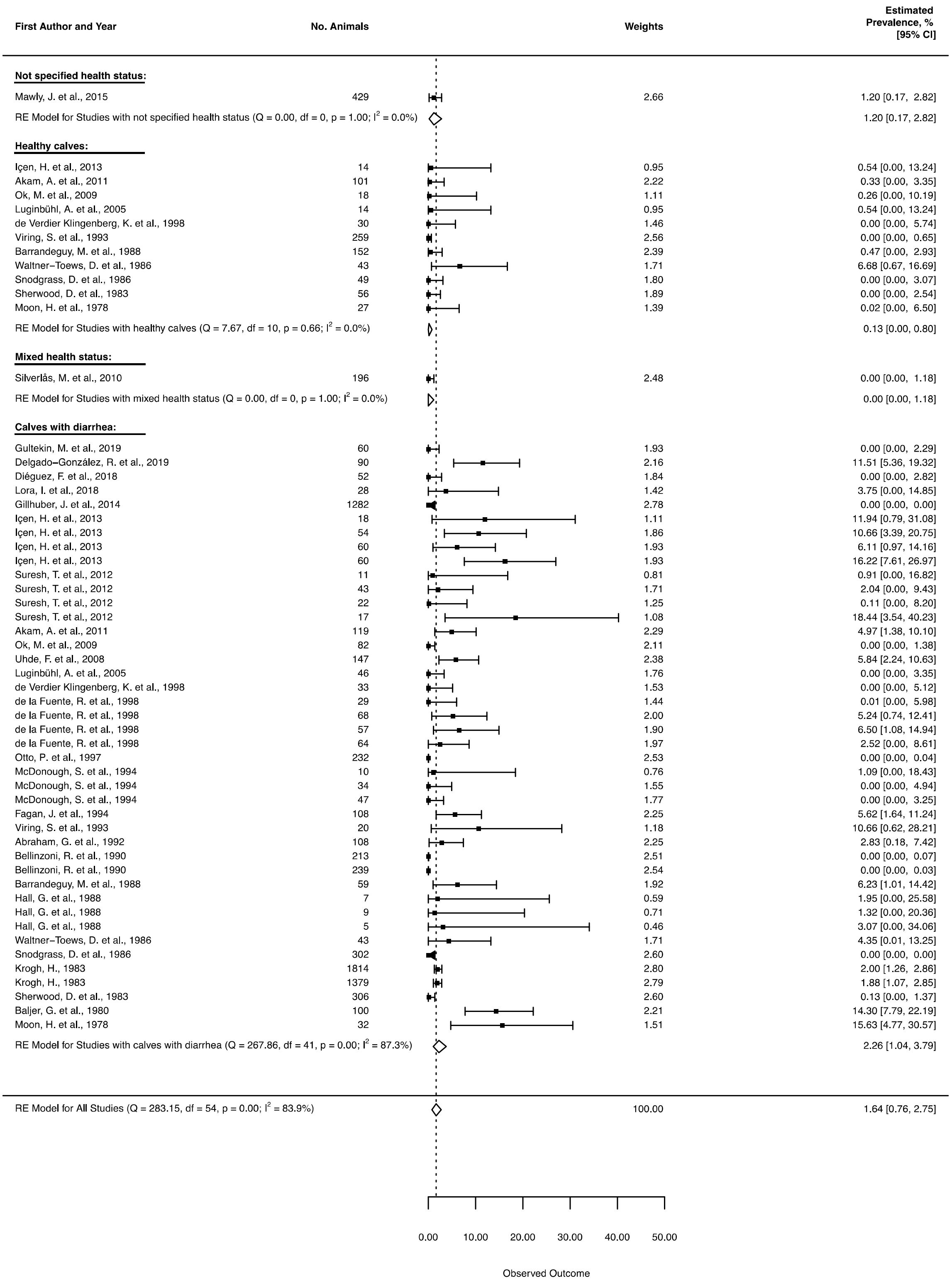

Fig. S4. Forest plot of studies with BRV-Crypto prevalences ordered by health status of the calves and publication year. N.B. The full references are provided at the end of

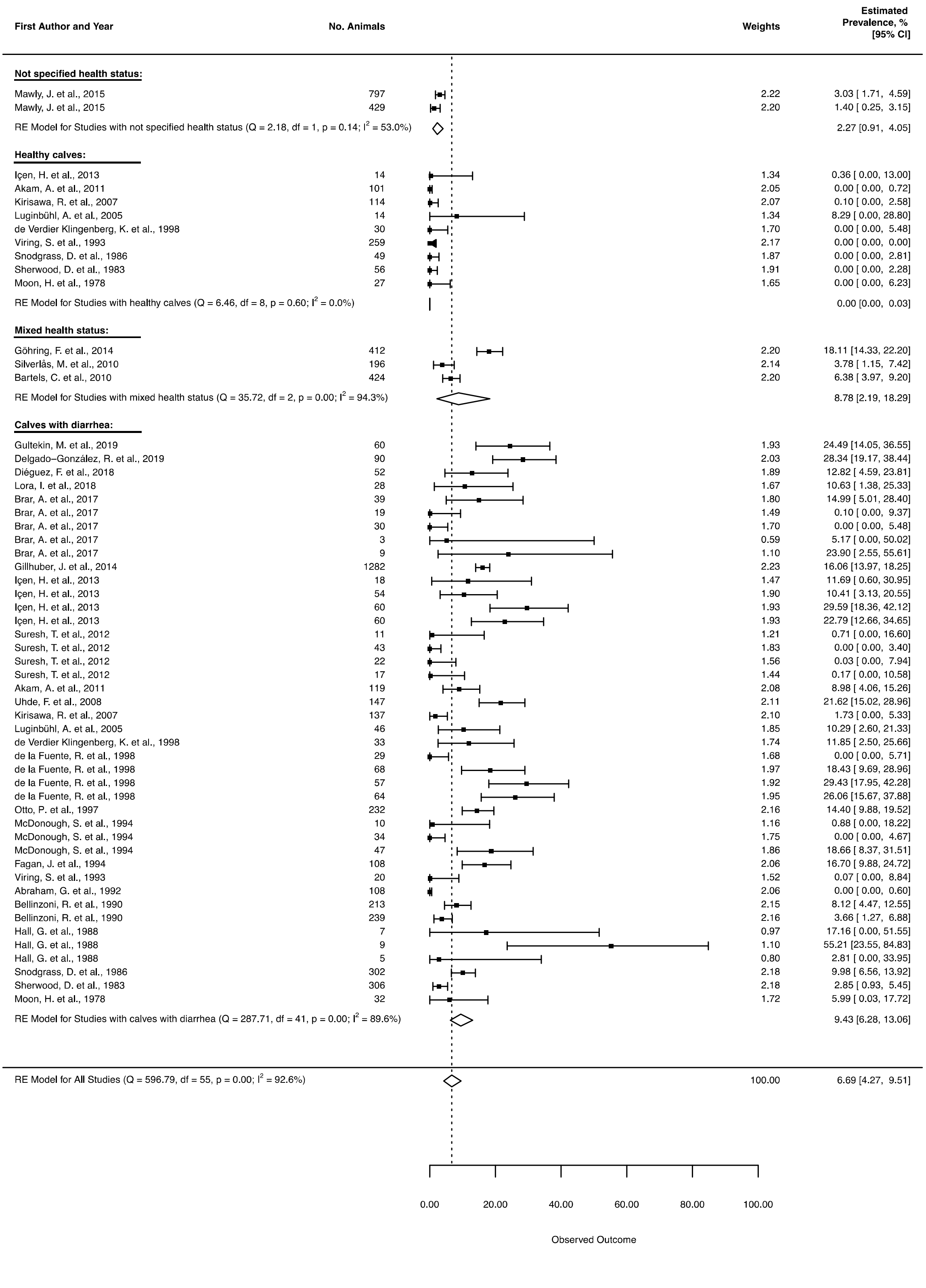

References of the 41 studies included in the Meta-analysis

(1) Abraham, G.; Roeder, P. L.; Zewdu, R. Agents associated with neonatal diarrhoea in Ethiopian dairy calves. *Trop. Anim. Health Prod.* **1992**, *24*, 74–80. https://doi.org/10.1007/BF02356948.

(2) Akam, A.; Khelef, D.; Kaidi, R.; Rahal, K.; Tali-Maamar, H.; Yabrir, B.; Laoun, A.; Mostfaoui, A.; Boutaiba, S.; Cozma, V. The frequency of the shedding of Cryptosporidium parvum, F5 Escherichia coli, rotavirus, coronavirus and Salmonella spp. in young dairy calves in Mitidja area (Algeria). *Bull. Univ. Agric. Sci. Vet. Med. Cluj-Napoca. Vet. Med.* **2011**, *68*, 16–25.

(3) Al Mawly, J.; Grinberg, A.; Prattley, D.; Moffat, J.; French, N. Prevalence of endemic enteropathogens of calves in New Zealand dairy farms. *N. Z. Vet. J.* **2015**, *63*, 147–152. https://doi.org/10.1080/00480169.2014.966168.

(4) Ammar, S. S. M.; Mokhtaria, K.; Tahar, B. B.; Amar, A. A.; Redha, B. A.; Yuva, B.; Mohamed, H. S.; Abdellatif, N.; Laid, B. Prevalence of rotavirus (GARV) and coronavirus (BCoV) associated with neonatal diarrhea in calves in western Algeria. *Asian Pac. J. Trop. Biomed.* **2014**, *4*, S318–S322. https://doi.org/10.12980/APJTB.4.2014C778.

(5) Baljer, G.; Bachmann, P. A. Demonstration of enteropathogenic Escherichia coli strains and rotaviruses in fecal samples from calves with diarrhea. *Zentralblatt für Veterinärmedizin B* **1980**, *27*, 608–615.

(6) Barrandeguy, M. E.; Cornaglia, E. M.; Gottschalk, M.; Fijtman, N.; Pasini, M. I.; Gómez Yafal, A.; Parraud, J. R.; Schudel, A. A. Rotavirus, enterotoxigenic Escherichia coli and other agents in the feces of dairy calves with and without diarrhea. *Rev. Latinoam. Microbiol.* **1988**, *30*, 239–245.

(7) Bartels, C. J. M.; Holzhauer, M.; Jorritsma, R.; Swart, W. A. J. M.; Lam, T. J. G. M. Prevalence, prediction and risk factors of enteropathogens in normal and non-normal faeces of young Dutch dairy calves. *Prev. Vet. Med.* **2010**, *93*, 162–169. https://doi.org/10.1016/j.prevetmed.2009.09.020.

(8) Bellinzoni, R. C.; Blackhall, J.; Terzolo, H. R.; Moreira, A. R.; Auza, N.; Mattion, N.; Micheo, G. L.; La Torre, J. L.; Scodeller, E. A. Microbiology of diarrhoea in young beef and dairy calves in Argentina. *Rev. Argent. Microbiol.* **1990**, *22*, 130–136.

(9) Brar, A. P. S.; Sood, N. K.; Kaur, P.; Singla, L. D.; Sandhu, B. S.; Gupta, K.; Narang, D.; Singh, C. K.; Chandra, M. Periurban outbreaks of bovine calf scours in Northern India caused by Cryptosporidium in association with other enteropathogens. *Epidemiol. Infect.* **2017**, *145*, 2717–2726. https://doi.org/10.1017/S0950268817001224.

(10) De la Fuente, R.; Garcıa, A.; Ruiz-Santa-Quiteria, J. A.; Luzon, M.; Cid, D.; Garcı́a, S.; Orden, J. A.; Gomez-Bautista, M. Proportional morbidity rates of enteropathogens among diarrheic dairy calves in central Spain. *Prev. Vet. Med.* **1998**, *36*, 145–152. https://doi.org/10.1016/s0167-5877(98)00077-4.

(11) de Verdier Klingenberg, K.; Svensson, L. Group A rotavirus as a cause of neonatal calf enteritis in Sweden. *Acta Vet. Scand.* **1998**, *39*, 195–199. https://doi.org/10.1186/BF03547792.
(12) Delgado-González, R. A.; Meza-Herrera, C. A.; González-Álvarez, V. H.; Alvarado-Espino, A. S.; Contreras-Villareal, V.; Gaytán-Alemán, L. R.; Arellano-Rodríguez, G.; Véliz-Deras, F. G. Enteropathogens in Holstein calves with diarrhea during the first five weeks of age in México. *Indian J. Anim. Res.* **2019**, *53*, 1085–1089.
(13) Dieguez, F. J.; González, A. M.; Soilán, M.; Eiras, C.; Sanjuán, M. L.; Yus, E. Evaluation of immunochromatographic test strips for rapid diagnosis of neonatal calf diarrhoea. *Cattle Pract.* **2012**, *20*, 1–4.
(14) Duckmanton, L.; Carman, S.; Nagy, É.; Petric, M. Detection of bovine torovirus in fecal specimens of calves with diarrhea from Ontario farms. *J. Clin. Microbiol.* **1998**, *36*, 1266–1270. https://doi.org/10.1128/JCM.36.5.1266-1270.1998.
(15) Fagan, J. G.; Dwyer, P. J.; Quinlan, J. F. The diagnosis and occurrence of enteropathogens associated with calf diarrhea in Ireland. *Ir. Vet. J.* **1994**, *47*, 313–318.
(16) Gillhuber, J.; Rügamer, D.; Pfister, K.; Scheuerle, M. C. Giardiosis and other enteropathogenic infections: A study on diarrhoeic calves in Southern Germany. *BMC Res. Notes* **2014**, *7*, 1–9. https://doi.org/10.1186/1756-0500-7-112.
(17) Goehring, F.; Moeller-Holtkamp, P.; Daugschies, A.; Lendner, M. Co-infections with Cryptosporidium parvum and other enteropathogenes support the occurrence and severity of diarrhoea in suckling calves. *Tierarztl. Umsch.* **2014**, *69*, 112–120.
(18) Gultekin, M.; Voyvoda, H.; Ural, K.; Erdogan, H.; Balikci, C.; Gultekin, G. Plasma citrulline, arginine, nitric oxide, and blood ammonia levels in neonatal calves with acute diarrhea. *J. Vet. Intern. Med.* **2019**, *33*, 987–998. https://doi.org/10.1111/jvim.15459.
(19) Hall, G. A.; Reynolds, D. J.; Parsons, K. R.; Bland, A. P.; Morgan, J. H. Pathology of calves with diarrhoea in southern Britain. *Res. Vet. Sci.* **1988**, *45*, 240–250. https://doi.org/10.1016/S0034-5288(18)30939-1.
(20) Hamann, H. P.; Herbst, W.; Krauss, H. Comparative investigation of the latex agglutination test" Slidex Rota-Kit 2" and electron microscopy for detection of rotavirus in fecal samples of calves with diarrhoea. *Berl. Munch. Tierarztl. Wochenschr.* **1989**, *102*, 346–347.
(21) Içen, H.; Arserim, N. B.; IŞIK, N.; Özkan, C.; Kaya, A. Prevalence of Four Enteropathogens with Immunochromatographic Rapid Test in the Feces of Diarrheic Calves in East and Southeast of Turkey. *Pak. Vet. J.* **2013**, *33*, 496–499.
(22) Kirisawa, R.; Takeyama, A.; Koiwa, M.; Iwai, H. Detection of bovine torovirus in fecal specimens of calves with diarrhea in Japan. *J. Vet. Med. Sci.* **2007**, *69*, 471–476. https://doi.org/10.1292/jvms.69.471.
(23) Krogh, H. V. Infection with enterotoxigenic Escherichia coli in calves and protection of the calves by vaccination of the dams. *Ann. Rech. Vétérinaires* **1983**, *14*, 522–525.

(24) Krogh, H. V. Occurrence of enterotoxigenic Escherichia coli in calves with acute neonatal diarrhoea. *Nord. Vet. Med.* **1983**, *35*, 346–352.
(25) Lanz Uhde, F.; Kaufmann, T.; Sager, H.; Albini, S.; Zanoni, R.; Schelling, E.; Meylan, M. Prevalence of four enteropathogens in the faeces of young diarrhoeic dairy calves in Switzerland. *Vet. Rec.* **2008**, *163*, 362–366. https://doi.org/10.1136/vr.163.12.362.
(26) Lora, I.; Gottardo, F.; Contiero, B.; Ava, B. D.; Bonfanti, L.; Stefani, A.; Barberio, A. Association between passive immunity and health status of dairy calves under 30 days of age. *Prev. Vet. Med.* **2018**, *152*, 12–15. https://doi.org/10.1016/j.prevetmed.2018.01.009.
(27) Lotthammer, K. H.; Ehlers, J. Epidemiological investigations on the frequencies of different viruses in calf losses in the Weser-Ems region. *Dtsch. Tierarztl. Wochenschr.* **1990**, *97*, 418–420.
(28) Luginbühl, A.; Reitt, K.; Metzler, A.; Kollbrunner, M.; Corboz, L.; Deplazes, P. Field study of the prevalence and diagnosis of diarrhea-causing agents in the newborn calf in a Swiss veterinary practice area. *Schweiz. Arch. Tierheilkd.* **2005**, *147*, 245–252. https://doi.org/10.1024/0036-7281.147.6.245.
(29) Mayameei, A.; Mohammadi, G.; Yavari, S.; Afshari, E.; Omidi, A. Evaluation of relationship between Rotavirus and Coronavirus infections with calf diarrhea by capture ELISA. *Comp. Clin. Path.* **2010**, *19*, 553–557. https://doi.org/10.1007/s00580-009-0920-x.
(30) McDonough, S. P.; Stull, C. L.; Osburn, B. I. Enteric pathogens in intensively reared veal calves. *Am. J. Vet. Res.* **1994**, *55*, 1516–1520.
(31) Moon, H. W.; McClurkin, A. W.; Isaacson, R. E.; Pohlenz, J.; Skartvedt, S. M.; Gillette, K. G.; Baetz, A. L. Pathogenic relationships of rotavirus, Escherichia coli, and other agents in mixed infections in calves. *J. Am. Vet. Med. Assoc.* **1978**, *173*, 577–583.
(32) Ok, M.; Güler, L.; Turgut, K.; Ok, Ü.; Şen, I.; Gündüz, I. K.; Birdane, M. F.; Güzelbekteş, H. The studies on the aetiology of diarrhoea in neonatal calves and determination of virulence gene markers of Escherichia coli strains by multiplex PCR. *Zoonoses Public Health* **2009**, *56*, 94–101. https://doi.org/10.1111/j.1863-2378.2008.01156.x.
(33) Otto, P.; Gunther, H.; Prudlo, J.; Godat, M. Calf diarrhoea: results and problems in diagnosing enteropathogens. *Tierarztl. Umsch.* **1997**, *52*, 563–568.
(34) Schlerka, G.; Gutler, S.; Baumgartner, W. Studies of aetiology, clinical signs, laboratory findings and treatment of milk-fed calves. *Tierarztl. Umsch.* **2002**, *57*, 189–194.
(35) Sherwood, D.; Snodgrass, D. R.; Lawson, G. H. Prevalence of enterotoxigenic Escherichia coli in calves in Scotland and northern England. *Vet. Rec.* **1983**, *113*, 208–212. https://doi.org/10.1136/vr.113.10.208.
(36) Silverlås, C.; De Verdier, K.; Emanuelson, U.; Mattsson, J. G.; Björkman, C. Cryptosporidium infection in herds with and without calf diarrhoeal problems. *Parasitol. Res.* **2010**, *107*, 1435–1444. https://doi.org/10.1007/s00436-010-2020-x.

(37) Snodgrass, D. R. R.; Terzolo, H. R.; Sherwood, D.; Campbell, I.; Menzies, J. D.; Synge, B. A. Aetiology of diarrhoea in young calves. *Vet. Rec.* **1986**, *119*, 31–34. https://doi.org/10.1136/vr.119.2.31.
(38) Suresh, T.; Rai, R. B.; Dhama, K.; Bhatt, P.; Sawant, P. M.; Sharma, A. K. Prevalence of rotavirus, coronavirus and Escherichia coli: the main agents responsible for calf diarrhoea. *Vet. Pract.* **2012**, *13*, 160–165.
(39) Vanamayya, P. R.; Mohanty, G. C.; Kumar, R.; Rao, D. R. Pathoepizootiological studies on viral enteritis/pneumoenteritis in neonatal calves. *Indian J. Anim. Sci.* **1993**, *63*, 391–397.
(40) Viring, S.; Olsson, S.-O.; Alenius, S.; Emanuelsson, U.; Jacobsson, S.-O.; Larsson, B.; Linde, N.; Uggla, A. Studies of enteric pathogens and gamma-globulin levels of neonatal calves in Sweden. *Acta Vet. Scand.* **1993**, *34*, 271–279.
(41) Waltner-Toews, D.; Martin, S. W.; Meek, A. H. An epidemiological study of selected calf pathogens on Holstein dairy farms in southwestern Ontario. *Can. J. Vet. Res.* **1986**, *50*, 307–313.
